# Late-Onset X-linked Adrenoleukodystrophy: A Rare Cause of Progressive Spastic Paraparesis

**DOI:** 10.7759/cureus.99429

**Published:** 2025-12-17

**Authors:** Sofia Sequeira, André Costa, Mariana Vargas, Ana Velon

**Affiliations:** 1 Internal Medicine, Hospital de Santo Espírito da Ilha Terceira, Angra do Heroísmo, PRT; 2 Neurology, Unidade Local de Saúde de Trás-os-Montes e Alto Douro, Lamego, PRT

**Keywords:** abcd1, adrenomyeloneuropathy, spastic paraparesis, very-long-chain fatty acids, x-linked adrenoleukodystrophy

## Abstract

X-linked adrenoleukodystrophy (X-ALD) is an uncommon peroxisomal disorder that can manifest in adult women with slowly progressive motor symptoms that often mimic hereditary spastic paraplegia, contributing to delayed diagnosis. We report the case of a 64-year-old woman with a long history of worsening gait impairment who had previously undergone spinal surgery without clinical benefit. She exhibited a spastic paraparesis with upper motor neuron features, and neuroimaging demonstrated white matter abnormalities in the brain with no structural explanation in the spine. Extensive laboratory testing excluded infectious, autoimmune, and metabolic causes. Biochemical evaluation revealed elevated very-long-chain fatty acids, raising suspicion for X-ALD, and genetic testing confirmed a heterozygous ABCD1 c.1849C>T (p.R617C) pathogenic variant. This case highlights the need to consider X-ALD in women presenting with unexplained progressive spastic paraparesis, emphasizing the value of timely biochemical and genetic evaluation to achieve an accurate diagnosis and provide appropriate guidance for affected families.

## Introduction

Progressive spastic paraparesis with adult onset is a clinical syndrome with a broad differential diagnosis, requiring comprehensive clinical, imaging, and laboratory investigation. It most often results from spinal cord pathology and is characterized by upper motor neuron signs (pyramidal syndrome), variable sensory deficits, and autonomic dysfunction involving bladder, sexual, and bowel control [1].

The underlying causes include extradural, intradural, and intramedullary lesions. Spinal magnetic resonance imaging (MRI) may be normal or may show spinal cord atrophy [1]. Common etiologies encompass immune-mediated, infectious, vascular, congenital, toxic-metabolic, degenerative, and neoplastic disorders.

Among toxic-metabolic causes, hereditary leukodystrophies are of particular relevance, with X-linked adrenoleukodystrophy (X-ALD) representing a key diagnostic consideration [2]. X-ALD is a rare genetic disorder caused by mutations in the ABCD1 gene located on Xq28, which encodes the peroxisomal adrenoleukodystrophy protein (ALDP). Deficiency of this protein leads to the accumulation of very-long-chain fatty acids in tissues dependent on peroxisomal metabolism, including the central nervous system white matter and adrenal cortex [3,4].

Although X-ALD predominantly affects males, heterozygous women may develop late-onset, slowly progressive neurological manifestations, typically emerging after the fourth or fifth decade of life, often manifesting as spastic paraparesis clinically indistinguishable from idiopathic forms, making diagnosis particularly challenging [5]. Detailed clinical assessment, family history, and biochemical and genetic testing are essential for establishing a definitive diagnosis [6].

## Case presentation

A 64-year-old woman with no significant past medical history presented with a five-year history of progressive gait disturbance. She reported dragging her left leg, gait imbalance, and occasional falls. Initial evaluation with lumbar spine MRI and electromyography (EMG) identified an L5-S1 disc herniation with corresponding radicular involvement. She underwent surgical decompression with partial improvement of radicular pain. Despite this, her gait gradually deteriorated over the following years, and she began requiring intermittent assistance for ambulation, along with increasing subjective lower-limb weakness.

Neurological examination revealed spastic paraparesis graded 4/5 on the Medical Research Council (MRC) scale, brisk lower-limb deep tendon reflexes (4+), bilateral Achilles clonus, bilateral Babinski sign, and a spastic-paretic gait pattern. Superficial and deep sensation remained intact. The remainder of the neurological examination was unremarkable. General physical examination showed long-standing diffuse alopecia, a weight of 47 kg, a height of 1.56 m, a blood pressure of 97/60 mmHg, and a heart rate of 68 bpm. Cardiopulmonary auscultation was normal, and there was no cutaneous or mucosal hyperpigmentation.

In recent months, the patient reported cognitive and memory difficulties that interfered with daily functioning. Family history was negative for neurological disorders, except for a cousin diagnosed with hereditary spastic paraplegia type 4 (SPG4). Given this family history, hereditary spastic paraplegia was considered a relevant differential diagnosis; however, genetic testing for SPG4 was negative, reducing the likelihood of this etiology.

**Figure 1:**
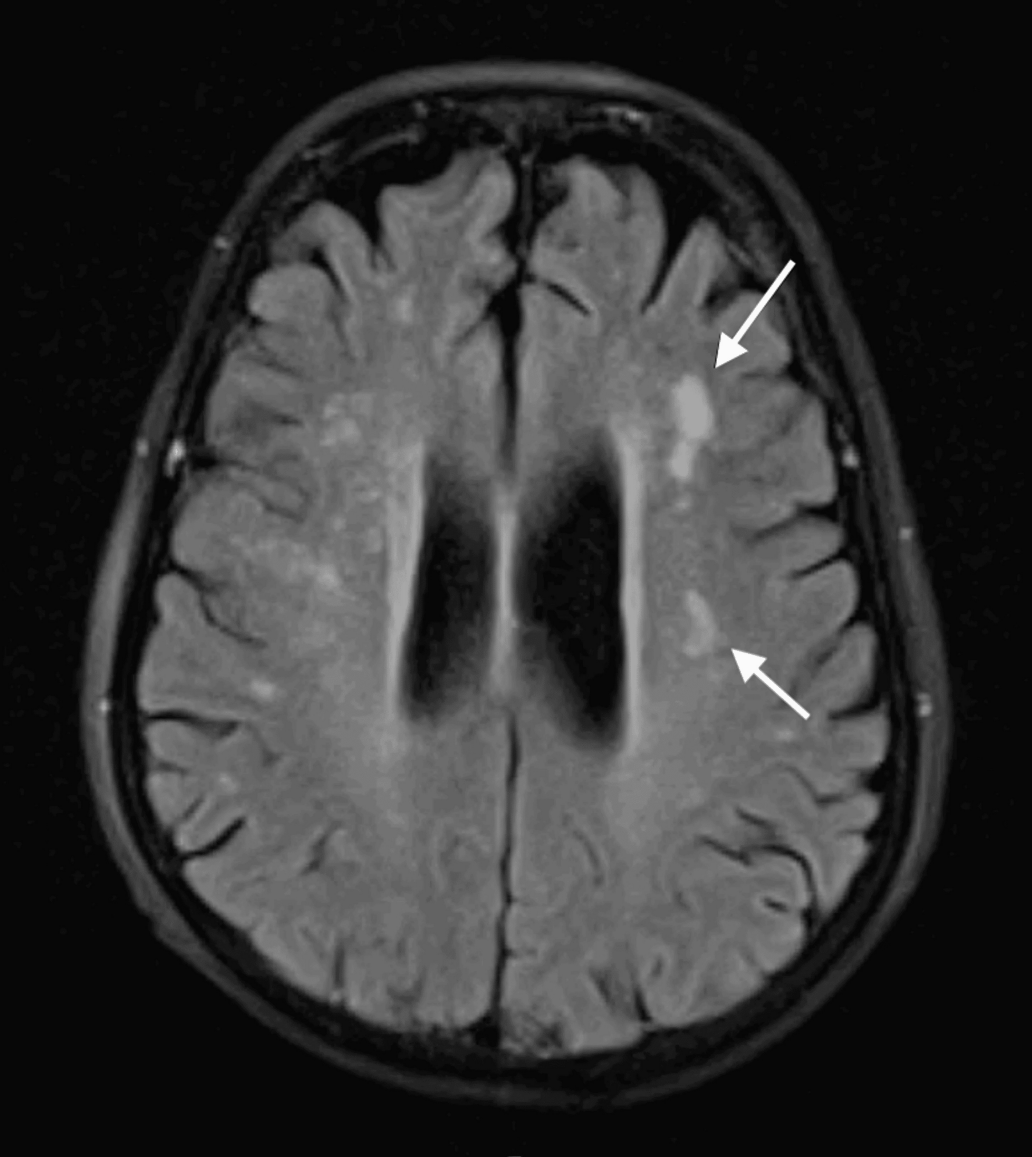
Brain MRI showing asymmetric, non-confluent supratentorial white matter hyperintensities without diffusion restriction, predominantly involving the periventricular regions MRI: magnetic resonance imaging

**Figure 2:**
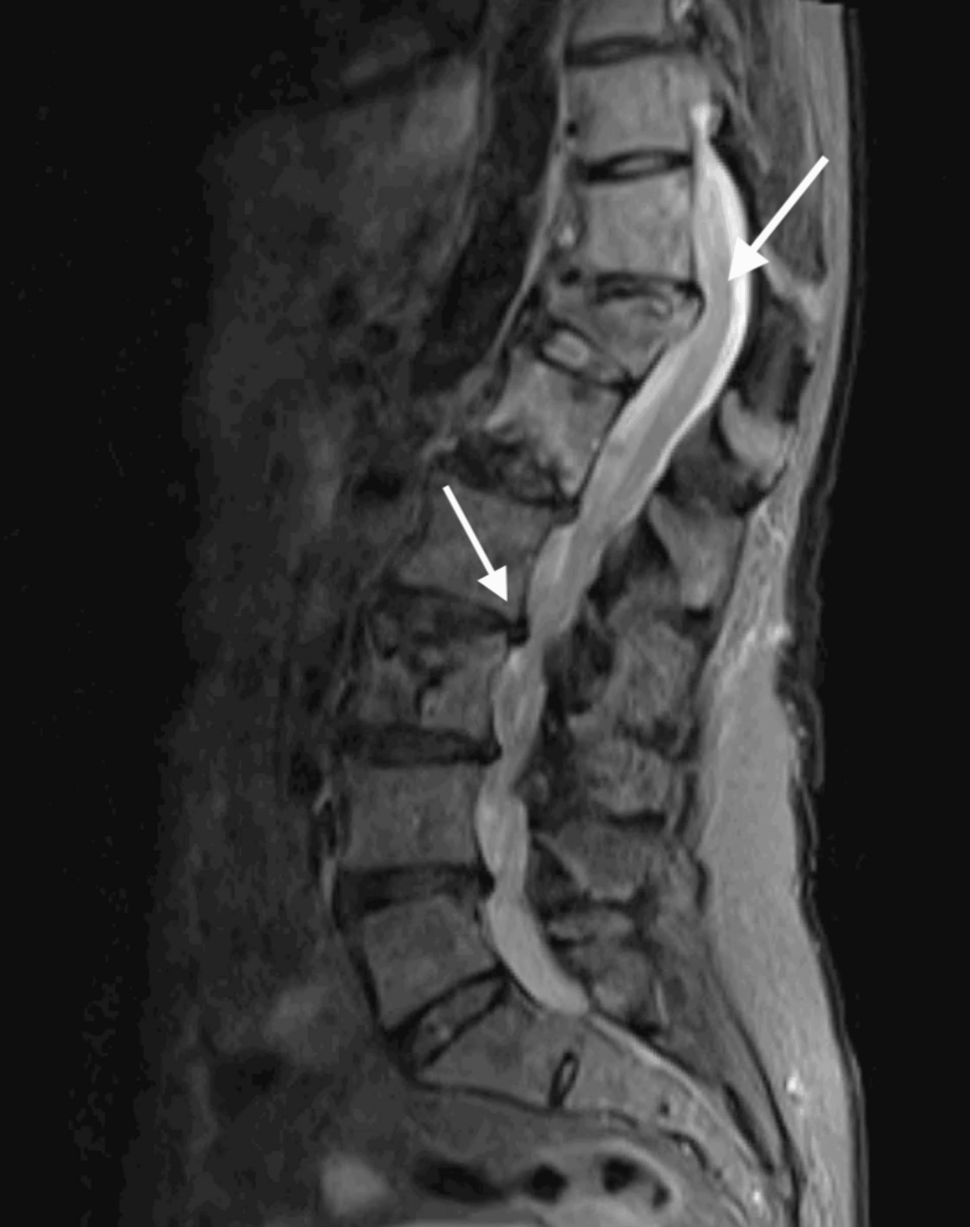
Sagittal T2-weighted spinal MRI showing multilevel degenerative changes without evidence of myelopathy MRI: magnetic resonance imaging

Brain MRI demonstrated supratentorial white matter hyperintensities, predominantly in the periventricular regions and left centrum semiovale. The lesions were asymmetric and non-confluent, and no diffusion restriction was observed on diffusion-weighted imaging (DWI)/apparent diffusion coefficient (ADC) sequences (Figure 1). Spinal MRI revealed multilevel degenerative changes without evidence of myelopathy (Figure 2).

EMG demonstrated severe distal neurogenic atrophy in the lower limbs. Routine laboratory tests, including complete blood count, metabolic panel, folate, vitamin B12, copper, angiotensin-converting enzyme, erythrocyte sedimentation rate, immunologic profile, thyroid and parathyroid function, adrenocorticotropic hormone (ACTH), and plasma cortisol, were within normal limits. Serologies for brucellosis, syphilis, HIV-1/HIV-2, and HTLV were negative. Cerebrospinal fluid (CSF) analysis was unremarkable.

Genetic testing for SPG4 was negative. Due to the progressive spastic phenotype, a peroxisomal biochemical evaluation was performed and demonstrated elevated very-long-chain fatty acids. Subsequent dried blood spot testing confirmed increased C26:0-lysophosphatidylcholine levels. 

The results are summarized in Table 1.

**Table 1:** Peroxisomal biochemical testing DBS: dried blood spot

Test	Result	Units	Reference range
C26:0	2.03	µmol/mL	0.16-0.57
C24:0/C22:0 ratio	1.82	-	0.63-1.10
C26:0/C22:0 ratio	0.137	-	0.004-0.022
C26:0-lysophosphatidylcholine (DBS)	0.71	µmol/L	0-0.10

Sequencing of the ABCD1 gene identified a heterozygous c.1849C>T (p.R617C) pathogenic variant, establishing the diagnosis of X-ALD. This variant has been previously reported in individuals with X-ALD and is associated with a broad phenotypic spectrum, ranging from adrenomyeloneuropathy (AMN) in men to slowly progressive myelopathic symptoms in heterozygous women.

The patient was also evaluated by Endocrinology, where adrenal insufficiency was excluded. She continues multidisciplinary follow-up in Neurology and Endocrinology, with partial stabilization of motor symptoms.

## Discussion

This case describes a late-onset heterozygous form of X-ALD presenting as slowly progressive spastic paraparesis in a 64-year-old woman, without evidence of adrenal involvement. This phenotype represents the most common clinical expression in female carriers of ABCD1 pathogenic variants, in whom demyelination and axonal degeneration progress insidiously over decades [5]. Overall, the clinical presentation in this patient is most consistent with a myelopathic, AMN-like phenotype, which is the predominant manifestation in heterozygous women. Although she reported mild cognitive symptoms, neuroimaging did not demonstrate features typical of cerebral adrenoleukodystrophy, and the findings do not suggest concomitant neurodegeneration beyond the expected X-ALD spectrum.

Adult-onset spastic paraparesis encompasses a broad differential diagnosis, including structural, infectious, inflammatory, vascular, and genetic causes [1]. In this patient, cervical, thoracic, and lumbar spinal MRI failed to demonstrate compressive or inflammatory myelopathy, and extensive laboratory evaluation excluded infectious etiologies such as HIV, HTLV, syphilis, and brucellosis, as well as nutritional deficiencies such as vitamin B12 or copper. This constellation of findings redirected the investigation toward a genetic-metabolic etiology.

Hereditary leukodystrophies should be considered in adults with progressive spastic paraparesis in the absence of a structural lesion [3,4]. Among these disorders, X-ALD is one of the most clinically relevant due to its prevalence and broad phenotypic spectrum, ranging from childhood cerebral forms to adult AMN [2]. In heterozygous women, 60-80% develop progressive neurological symptoms after the fourth decade of life, typically manifesting as a spastic paraparesis clinically indistinguishable from hereditary spastic paraplegia, often leading to diagnostic delays of several years [5].

X-ALD results from mutations in the ABCD1 gene, which encodes the peroxisomal ALDP. Dysfunction of ALDP impairs the transport and degradation of very-long-chain fatty acids, leading to their accumulation in the central nervous system, spinal cord, and adrenal cortex [7]. Measurement of elevated plasma very-long-chain fatty acids, particularly C26:0 and the C24:0/C22:0 and C26:0/C22:0 ratios, remains a key biochemical diagnostic marker [8]. More recently, C26:0-lysophosphatidylcholine in dried blood spots has shown excellent diagnostic performance and is elevated in nearly all affected females [9]. However, very-long-chain fatty acid levels may be normal in approximately 10-20% of heterozygous women, contributing to underdiagnosis and underscoring the importance of genetic testing when clinical suspicion persists [9]. In this case, both biochemical abnormalities and the identification of a pathogenic ABCD1 variant confirmed the diagnosis of X-ALD. The ABCD1 p.R617C variant identified in this patient has been described in prior reports and is associated with heterogeneous phenotypes, consistent with the well-known lack of strict genotype-phenotype correlation in X-ALD.

The absence of adrenal insufficiency in this patient is consistent with the typical endocrine phenotype of heterozygous women, although periodic endocrine surveillance is recommended as adrenal involvement may appear later in life [7]. Chronic alopecia in this patient is likely an incidental finding. Although peroxisomal disorders can occasionally present with cutaneous manifestations, any association between alopecia and X-ALD remains speculative and unsupported by current evidence.

There is currently no curative therapy for the myelopathic form of X-ALD (AMN phenotype), and management relies on supportive care and rehabilitation to maintain mobility and quality of life. In contrast, early cerebral involvement may benefit from hematopoietic stem cell transplantation (HSCT) or, more recently, hematopoietic stem cell gene therapy with elivaldogene autotemcel, both of which can stabilize neurological progression when implemented at early disease stages [10,11].

This case highlights the importance of including X-ALD in the differential diagnosis of adult-onset progressive spastic paraparesis, particularly in women with suggestive family history or compatible biochemical profiles. Early recognition enables timely genetic counseling, screening of male relatives, and endocrine monitoring, interventions essential to improving long-term outcomes and preventing complications [2].

## Conclusions

X-ALD should be considered in the differential diagnosis of progressive spastic paraparesis of unclear etiology, including in adult heterozygous women in whom symptoms typically present later in life and progress slowly. Early recognition is essential to avoid diagnostic delays and to enable appropriate family genetic counseling, endocrine surveillance, and structured neurological follow-up.

This case underscores the importance of incorporating metabolic leukodystrophies into the evaluation of chronic myelopathies without an evident cause, emphasizing the diagnostic value of peroxisomal biochemical testing and ABCD1 genetic analysis. Non-classical findings, such as chronic alopecia in this patient, are likely incidental, and their clinical significance remains uncertain, warranting no definitive association with X-ALD at present.
